# Methyl 2-[4-(tri­fluoro­meth­yl)phenyl­sulfan­yl]benzoate

**DOI:** 10.1107/S1600536813028146

**Published:** 2013-10-19

**Authors:** Thammarse S. Yamuna, Jerry P. Jasinski, Brian J. Anderson, H. S. Yathirajan, Manpreet Kaur

**Affiliations:** a Department of Studies in Chemistry, University of Mysore, Manasagangotri, Mysore 570 006, India; b Department of Chemistry, Keene State College, 229 Main Street, Keene, NH 03435-2001, USA

## Abstract

In the title compound, C_15_H_13_F_3_O_2_S, the dihedral angle between the benzene rings is 79.5 (1)°. The ester group is twisted by 7.6 (1)° from the mean plane of the adjacent benzene ring. Disorder was modeled over two sites for one F atom of the tri­fluoro­methyl group with an occupancy ratio of 0.54 (6):0.46 (6). In the crystal, mol­ecules are linked *via* weak C—H⋯O hydrogen bonds, forming two-dimensional networks lying parallel to (101). The networks are linked *via* C—H⋯π inter­actions, leading to the formation of a three-dimensional supra­molecular structure.

## Related literature

For general background and pharmacological properties of the neuroleptic agent flupentixol [systematic name: *(EZ)*-2-[4-[3-[2-(tri­fluoro­meth­yl)thioxanthen-9-yl­idene]prop­yl]pip­era­zin-1-yl]ethanol] and related compounds, see: Ovhed (1976 ▶); Robertson & Trimble (1981 ▶); Valle-Jones & Swarbrick (1981 ▶); Young *et al.* (1976 ▶). For related structures, see: Post *et al.* (1975*a* ▶,*b* ▶); Siddegowda *et al.* (2011*a* ▶,*b* ▶). For standard bond lengths, see: Allen *et al.* (1987 ▶).
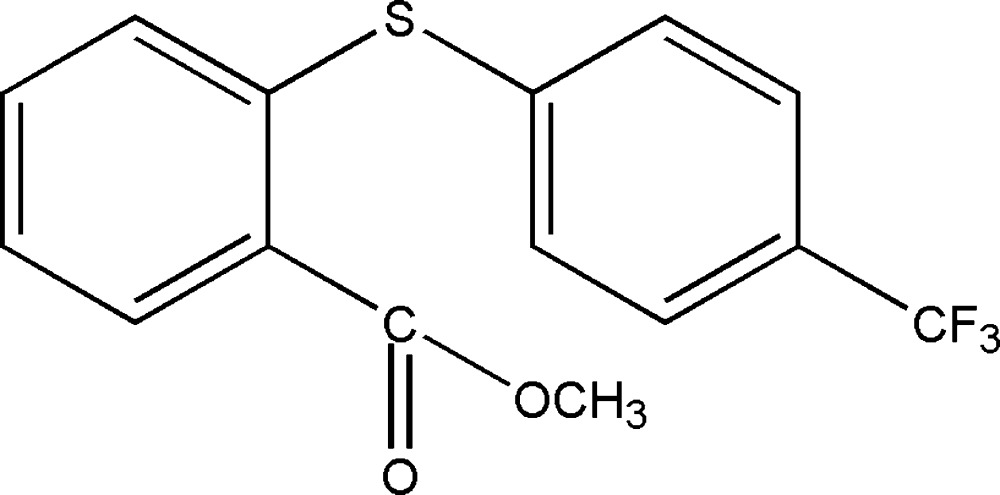


## Experimental

### 

#### Crystal data

C_15_H_11_F_3_O_2_S
*M* *_r_* = 312.30
Monoclinic, 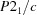

*a* = 11.0675 (5) Å
*b* = 8.0429 (3) Å
*c* = 15.6614 (7) Å
β = 96.654 (5)°
*V* = 1384.70 (11) Å^3^
*Z* = 4
Cu *K*α radiation
μ = 2.44 mm^−1^
*T* = 173 K
0.28 × 0.22 × 0.12 mm

#### Data collection

Agilent Gemini EOS diffractometer
Absorption correction: multi-scan (*CrysAlis PRO* and *CrysAlis RED*; Agilent, 2012 ▶) *T*_min_ = 0.715, *T*_max_ = 1.000
8110 measured reflections
2705 independent reflections
2224 reflections with *I* > 2σ(*I*)
*R*_int_ = 0.039

#### Refinement

*R*[*F* ^2^ > 2σ(*F* ^2^)] = 0.043
*wR*(*F* ^2^) = 0.123
*S* = 1.03
2705 reflections
201 parameters
H-atom parameters constrained
Δρ_max_ = 0.36 e Å^−3^
Δρ_min_ = −0.27 e Å^−3^

### 

Data collection: *CrysAlis PRO* (Agilent, 2012 ▶); cell refinement: *CrysAlis PRO*; data reduction: *CrysAlis RED* (Agilent, 2012 ▶); program(s) used to solve structure: *SUPERFLIP* (Palatinus & Chapuis, 2007 ▶); program(s) used to refine structure: *SHELXL2012* (Sheldrick, 2008 ▶); molecular graphics: *OLEX2* (Dolomanov *et al.*, 2009 ▶); software used to prepare material for publication: *OLEX2*.

## Supplementary Material

Crystal structure: contains datablock(s) I. DOI: 10.1107/S1600536813028146/su2658sup1.cif

Structure factors: contains datablock(s) I. DOI: 10.1107/S1600536813028146/su2658Isup2.hkl

Supplementary material file. DOI: 10.1107/S1600536813028146/su2658Isup3.cml

Additional supplementary materials:  crystallographic information; 3D view; checkCIF report

## Figures and Tables

**Table 1:** Hydrogen-bond geometry (Å, °) *Cg* is the centroid of the C2–C7 benzene ring.

*D*—H⋯*A*	*D*—H	H⋯*A*	*D*⋯*A*	*D*—H⋯*A*
C15—H15*C*⋯O2^i^	0.96	2.45	3.221 (3)	138
C12—H12⋯*Cg* ^ii^	0.93	2.70	3.558 (2)	154

Symmetry codes: (i) 
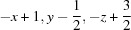
; (ii) 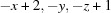.

**Fig. 1.**
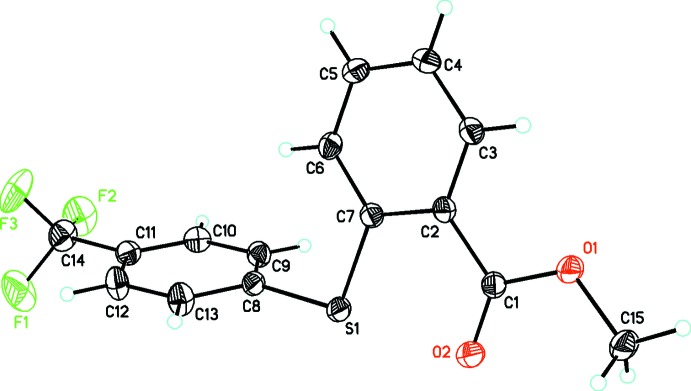
A view of the molecular structure of the title molecule, with atom labelling. Displacement ellipsoids are drawn at the 50% probability level [the disordered atom F3A, with occupancy 0.46 (6), has been removed for clarity].

**Fig. 2.**
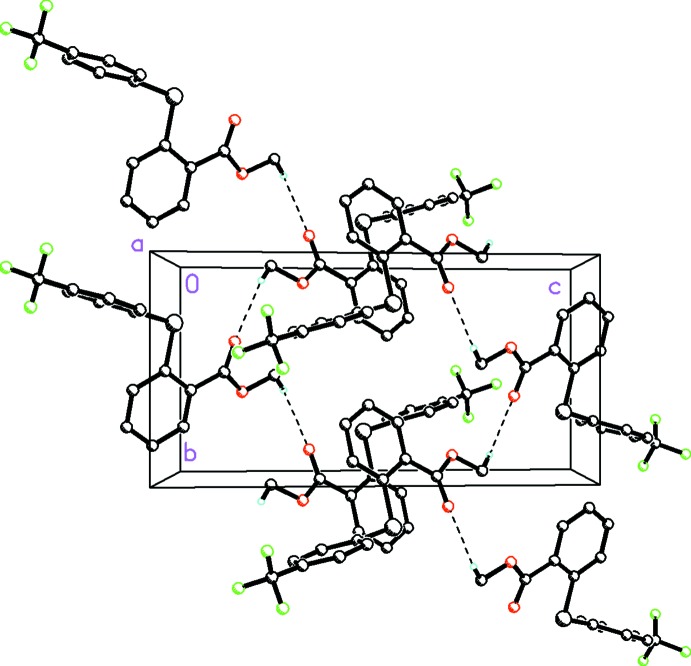
A viewed along the a axis of the crystal packing of the title compound. The hydrogen bonds are shown as dashed lines [see Table 1 for details; disordered atom F3A, and H atoms not involved in hydrogen bonding, have been omitted for clarity].

## supplementary crystallographic information

### 1. Comment

The title compound,C_15_H_13_F_3_O_2_S, is a methyl ester derivative of the starting material for the synthesis of the flupentixol [systematic name: *(EZ)*-2-[4-[3-[2-(trifluoromethyl)thioxanthen-9-ylidene]propyl]piperazin-1-yl]ethanol], a well documented neuroleptic. There have been many controlled studies that compared it with a placebo (Ovhed, 1976) and classical antidepressants (Young *et al.*, 1976). Low-dose neuroleptics have been applied increasingly in recent years to treat anxiety and depression (Robertson & Trimble, 1981; Valle-Jones & Swarbrick, 1981). The crystal structures of α-flupenthixol (Post *et al.*, 1975*a*) and β-flupenthixol (Post *et al.*, 1975*b*) have been reported. The crystal structures of some related compounds reported by our group are: 1-(2-Hydroxyethyl)-4-[3-(2-trifluoromethyl-9H-thioxanthen- 9-ylidene)propyl]piperazine-1,4-diium dichloride: the dihydrochloride salt of flupentixol (Siddegowda *et al.*, 2011*a*), and 1-(2-Hydroxyethyl) -4-{3-[(E)-2-(trifluoromethyl)-9H-thioxanthen-9-ylidene]propyl}piperazine-1,4-diium bis(3-carboxyprop-2-enoate) (Siddegowda *et al.*, 2011*b*). In view of the importance of flupentixol, we report herein on the crystal structure of the title compound a methyl ester derivative of the starting material for the synthesis of the flupentixol.

In the title molecule, Fig. 1, the dihedral angle between the two benzene rings, (C2-C7) and (C8-C13) is 79.5 (1)°. The ester group (O1/C1/C2/O2) is twisted by 7.6 (1)° from the mean plane of the adjacent benzene ring (C2–C7). Disorder was modeled over two sets of sites for fluorine atom, F3/F3A of the trifluoromethyl group, with an occupancy ratio of 0.54 (6) : 0.46 (6). Bond lengths are in normal ranges (Allen *et al.*, 1987).

In the crystal, molecules are linked via C—H···O hydrogen bonds forming two-dimensional networks lying parallel to (101). These networks are linked via C-H···π interactions leading to the formation of a three-dimensional supramolecular structure (Table 1 and Fig. 2).

### 2. Experimental

The reactant 2-(4-Trifluoromethylphenylsulfanyl)benzoic acid (I) was obtained as a gift sample from R. L. Fine Chem, Bengaluru, India. 10 g of (I) [0.0335 mol] was dissolved in 50 ml of methanol, followed by the addition of 1 ml of 98% sulphuric acid and the mixture was refluxed for 8 hours at 338 K. The methanol was distilled off and 100 ml of 10% Na_2_CO_3_ solution was added and the mixture stirred for 5 min. This was then extracted with dichloromethane and then the solvent was removed by distillation. The product formed was recrystallized from methanol giving colourless block-like crystals of the title compound (M.p. = 413-418 K).

### 3. Refinement

All of the H atoms were placed in their calculated positions and refined using the riding model approximation: C-H = 0.93 Å (CH) and 0.96 Å (CH_3_), with U_iso_(H) = 1.5_eq_(C-methyl) and = 1.2U_eq_(C) for other H atoms. Disorder for one fluorine atom (F3/F3A) in the trifluoromethyl group was modeled over two sets of sites with an occupancy ratio of 0.54 (6):0.46 (6).

### Crystal data

**Table:**

C_15_H_11_F_3_O_2_S	*F*(000) = 640
*M**_r_* = 312.30	*D*_x_ = 1.498 Mg m^−^^3^
Monoclinic, *P*2_1_/*c*	Cu *K*α radiation, λ = 1.54184 Å
*a* = 11.0675 (5) Å	Cell parameters from 2806 reflections
*b* = 8.0429 (3) Å	θ = 4.0–72.2°
*c* = 15.6614 (7) Å	µ = 2.44 mm^−^^1^
β = 96.654 (5)°	*T* = 173 K
*V* = 1384.70 (11) Å^3^	Block, colourless
*Z* = 4	0.28 × 0.22 × 0.12 mm

### Data collection

**Table:**

Agilent Gemini EOS diffractometer	2705 independent reflections
Radiation source: Enhance (Cu) X-ray Source	2224 reflections with *I* > 2σ(*I*)
Detector resolution: 16.0416 pixels mm^-1^	*R*_int_ = 0.039
ω scans	θ_max_ = 72.4°, θ_min_ = 4.0°
Absorption correction: multi-scan (*CrysAlis PRO* and *CrysAlis RED*; Agilent, 2012)	*h* = −13→10
*T*_min_ = 0.715, *T*_max_ = 1.000	*k* = −9→7
8110 measured reflections	*l* = −18→19

### Refinement

**Table:**

Refinement on *F*^2^	Primary atom site location: structure-invariant direct methods
Least-squares matrix: full	Hydrogen site location: inferred from neighbouring sites
*R*[*F*^2^ > 2σ(*F*^2^)] = 0.043	H-atom parameters constrained
*wR*(*F*^2^) = 0.123	*w* = 1/[σ^2^(*F*_o_^2^) + (0.0689*P*)^2^ + 0.2842*P*] where *P* = (*F*_o_^2^ + 2*F*_c_^2^)/3
*S* = 1.03	(Δ/σ)_max_ < 0.001
2705 reflections	Δρ_max_ = 0.36 e Å^−^^3^
201 parameters	Δρ_min_ = −0.27 e Å^−^^3^
0 restraints	

### Special details

**Table:**

Geometry. All esds (except the esd in the dihedral angle between two l.s. planes) are estimated using the full covariance matrix. The cell esds are taken into account individually in the estimation of esds in distances, angles and torsion angles; correlations between esds in cell parameters are only used when they are defined by crystal symmetry. An approximate (isotropic) treatment of cell esds is used for estimating esds involving l.s. planes.

### Fractional atomic coordinates and isotropic or equivalent isotropic displacement parameters (Å^2^)

**Table:**

	*x*	*y*	*z*	*U*_iso_*/*U*_eq_	Occ. (<1)
S1	0.74068 (5)	0.20434 (6)	0.53524 (4)	0.03547 (18)	
F1	1.15258 (18)	0.4950 (3)	0.29524 (12)	0.0867 (7)	
F2	1.02027 (17)	0.4280 (3)	0.19408 (10)	0.0764 (6)	
F3	1.1534 (11)	0.257 (2)	0.2621 (9)	0.078 (3)	0.54 (6)
F3A	1.121 (4)	0.234 (3)	0.240 (3)	0.127 (7)	0.46 (6)
O1	0.50339 (13)	−0.10252 (18)	0.67852 (9)	0.0370 (4)	
O2	0.61424 (16)	0.1251 (2)	0.66627 (11)	0.0504 (5)	
C1	0.58173 (17)	−0.0107 (3)	0.64017 (13)	0.0289 (4)	
C2	0.62526 (16)	−0.0927 (2)	0.56503 (12)	0.0268 (4)	
C3	0.59456 (18)	−0.2586 (3)	0.54673 (13)	0.0314 (4)	
H3	0.5458	−0.3151	0.5816	0.038*	
C4	0.63512 (19)	−0.3401 (3)	0.47797 (14)	0.0354 (5)	
H4	0.6149	−0.4509	0.4670	0.042*	
C5	0.70653 (19)	−0.2546 (3)	0.42526 (13)	0.0343 (5)	
H5	0.7332	−0.3082	0.3783	0.041*	
C6	0.73828 (18)	−0.0906 (3)	0.44206 (13)	0.0313 (4)	
H6	0.7866	−0.0355	0.4063	0.038*	
C7	0.69912 (16)	−0.0063 (2)	0.51164 (13)	0.0271 (4)	
C8	0.83570 (18)	0.2519 (2)	0.45386 (13)	0.0310 (4)	
C9	0.78756 (19)	0.2956 (3)	0.37134 (15)	0.0352 (5)	
H9	0.7037	0.2965	0.3566	0.042*	
C10	0.8636 (2)	0.3378 (3)	0.31074 (14)	0.0368 (5)	
H10	0.8312	0.3660	0.2552	0.044*	
C11	0.98830 (19)	0.3377 (3)	0.33316 (14)	0.0345 (5)	
C12	1.0368 (2)	0.2971 (3)	0.41589 (15)	0.0435 (6)	
H12	1.1205	0.2989	0.4310	0.052*	
C13	0.9604 (2)	0.2538 (3)	0.47609 (15)	0.0419 (5)	
H13	0.9929	0.2258	0.5317	0.050*	
C14	1.0745 (2)	0.3760 (3)	0.26912 (17)	0.0493 (6)	
C15	0.4586 (2)	−0.0262 (3)	0.75237 (14)	0.0406 (5)	
H15A	0.4236	0.0801	0.7363	0.061*	
H15B	0.5247	−0.0117	0.7972	0.061*	
H15C	0.3979	−0.0964	0.7727	0.061*	

### Atomic displacement parameters (Å^2^)

**Table:**

	*U*^11^	*U*^22^	*U*^33^	*U*^12^	*U*^13^	*U*^23^
S1	0.0389 (3)	0.0280 (3)	0.0428 (3)	−0.0061 (2)	0.0183 (2)	−0.0040 (2)
F1	0.0817 (12)	0.1103 (16)	0.0725 (12)	−0.0512 (12)	0.0279 (10)	0.0079 (11)
F2	0.0827 (12)	0.1077 (16)	0.0416 (9)	−0.0139 (11)	0.0196 (8)	0.0172 (9)
F3	0.078 (6)	0.083 (8)	0.083 (5)	0.030 (4)	0.054 (5)	0.025 (4)
F3A	0.196 (15)	0.049 (4)	0.166 (15)	0.017 (9)	0.151 (12)	0.011 (8)
O1	0.0469 (9)	0.0324 (8)	0.0350 (8)	−0.0067 (6)	0.0189 (7)	−0.0022 (6)
O2	0.0583 (10)	0.0422 (9)	0.0564 (11)	−0.0182 (8)	0.0304 (8)	−0.0184 (8)
C1	0.0261 (9)	0.0314 (10)	0.0295 (10)	−0.0011 (8)	0.0042 (7)	0.0000 (8)
C2	0.0229 (9)	0.0297 (10)	0.0277 (9)	−0.0004 (7)	0.0020 (7)	0.0010 (7)
C3	0.0304 (10)	0.0308 (10)	0.0335 (10)	−0.0052 (8)	0.0061 (8)	0.0007 (8)
C4	0.0392 (11)	0.0281 (10)	0.0391 (12)	−0.0053 (9)	0.0057 (9)	−0.0051 (8)
C5	0.0378 (11)	0.0345 (11)	0.0316 (10)	0.0019 (9)	0.0082 (9)	−0.0058 (8)
C6	0.0310 (10)	0.0327 (11)	0.0317 (10)	0.0014 (8)	0.0099 (8)	0.0034 (8)
C7	0.0249 (9)	0.0246 (9)	0.0320 (10)	0.0007 (7)	0.0036 (7)	0.0013 (7)
C8	0.0329 (10)	0.0251 (9)	0.0368 (11)	−0.0028 (8)	0.0117 (8)	0.0011 (8)
C9	0.0293 (10)	0.0331 (11)	0.0429 (12)	0.0018 (8)	0.0027 (9)	0.0012 (9)
C10	0.0433 (12)	0.0341 (11)	0.0325 (11)	0.0014 (9)	0.0031 (9)	0.0049 (9)
C11	0.0394 (11)	0.0300 (11)	0.0353 (11)	−0.0036 (9)	0.0098 (9)	0.0018 (8)
C12	0.0289 (11)	0.0611 (16)	0.0407 (13)	−0.0067 (10)	0.0046 (9)	0.0080 (11)
C13	0.0361 (12)	0.0558 (14)	0.0337 (11)	−0.0031 (10)	0.0039 (9)	0.0097 (10)
C14	0.0543 (15)	0.0508 (15)	0.0458 (14)	−0.0043 (12)	0.0186 (11)	0.0103 (11)
C15	0.0508 (13)	0.0427 (13)	0.0315 (11)	−0.0040 (10)	0.0179 (9)	−0.0010 (9)

### Geometric parameters (Å, º)

**Table:**

S1—C7	1.783 (2)	C5—C6	1.382 (3)
S1—C8	1.785 (2)	C6—H6	0.9300
F1—C14	1.322 (3)	C6—C7	1.394 (3)
F2—C14	1.324 (3)	C8—C9	1.385 (3)
F3—C14	1.311 (13)	C8—C13	1.384 (3)
F3A—C14	1.354 (19)	C9—H9	0.9300
O1—C1	1.334 (2)	C9—C10	1.382 (3)
O1—C15	1.446 (2)	C10—H10	0.9300
O2—C1	1.206 (3)	C10—C11	1.384 (3)
C1—C2	1.478 (3)	C11—C12	1.383 (3)
C2—C3	1.398 (3)	C11—C14	1.494 (3)
C2—C7	1.417 (3)	C12—H12	0.9300
C3—H3	0.9300	C12—C13	1.382 (3)
C3—C4	1.380 (3)	C13—H13	0.9300
C4—H4	0.9300	C15—H15A	0.9600
C4—C5	1.389 (3)	C15—H15B	0.9600
C5—H5	0.9300	C15—H15C	0.9600
			
C7—S1—C8	102.41 (9)	C10—C9—H9	119.9
C1—O1—C15	115.20 (17)	C9—C10—H10	120.2
O1—C1—C2	113.60 (17)	C9—C10—C11	119.6 (2)
O2—C1—O1	122.19 (19)	C11—C10—H10	120.2
O2—C1—C2	124.20 (18)	C10—C11—C14	121.7 (2)
C3—C2—C1	119.69 (18)	C12—C11—C10	120.3 (2)
C3—C2—C7	119.33 (18)	C12—C11—C14	118.0 (2)
C7—C2—C1	120.97 (18)	C11—C12—H12	120.1
C2—C3—H3	119.3	C13—C12—C11	119.8 (2)
C4—C3—C2	121.35 (19)	C13—C12—H12	120.1
C4—C3—H3	119.3	C8—C13—H13	119.9
C3—C4—H4	120.4	C12—C13—C8	120.1 (2)
C3—C4—C5	119.17 (19)	C12—C13—H13	119.9
C5—C4—H4	120.4	F1—C14—F2	105.0 (2)
C4—C5—H5	119.7	F1—C14—F3A	117 (2)
C6—C5—C4	120.58 (19)	F1—C14—C11	112.7 (2)
C6—C5—H5	119.7	F2—C14—F3A	97 (2)
C5—C6—H6	119.4	F2—C14—C11	113.7 (2)
C5—C6—C7	121.16 (19)	F3—C14—F1	98.0 (8)
C7—C6—H6	119.4	F3—C14—F2	113.3 (7)
C2—C7—S1	119.74 (15)	F3—C14—C11	112.8 (7)
C6—C7—S1	121.86 (15)	F3A—C14—C11	110.7 (8)
C6—C7—C2	118.40 (18)	O1—C15—H15A	109.5
C9—C8—S1	121.72 (16)	O1—C15—H15B	109.5
C13—C8—S1	118.39 (16)	O1—C15—H15C	109.5
C13—C8—C9	119.78 (19)	H15A—C15—H15B	109.5
C8—C9—H9	119.9	H15A—C15—H15C	109.5
C10—C9—C8	120.3 (2)	H15B—C15—H15C	109.5
			
S1—C8—C9—C10	177.55 (16)	C8—S1—C7—C6	−1.42 (18)
S1—C8—C13—C12	−177.2 (2)	C8—C9—C10—C11	−0.6 (3)
O1—C1—C2—C3	−7.3 (3)	C9—C8—C13—C12	−0.8 (4)
O1—C1—C2—C7	173.53 (17)	C9—C10—C11—C12	−0.5 (3)
O2—C1—C2—C3	171.6 (2)	C9—C10—C11—C14	177.6 (2)
O2—C1—C2—C7	−7.6 (3)	C10—C11—C12—C13	1.0 (4)
C1—C2—C3—C4	−178.95 (19)	C10—C11—C14—F1	127.0 (3)
C1—C2—C7—S1	0.1 (2)	C10—C11—C14—F2	7.6 (4)
C1—C2—C7—C6	179.48 (17)	C10—C11—C14—F3	−123.1 (8)
C2—C3—C4—C5	−0.9 (3)	C10—C11—C14—F3A	−100 (2)
C3—C2—C7—S1	−179.11 (15)	C11—C12—C13—C8	−0.4 (4)
C3—C2—C7—C6	0.3 (3)	C12—C11—C14—F1	−54.9 (3)
C3—C4—C5—C6	1.0 (3)	C12—C11—C14—F2	−174.2 (2)
C4—C5—C6—C7	−0.4 (3)	C12—C11—C14—F3	55.0 (9)
C5—C6—C7—S1	179.17 (16)	C12—C11—C14—F3A	78 (2)
C5—C6—C7—C2	−0.2 (3)	C13—C8—C9—C10	1.3 (3)
C7—S1—C8—C9	81.16 (19)	C14—C11—C12—C13	−177.2 (2)
C7—S1—C8—C13	−102.49 (19)	C15—O1—C1—O2	1.2 (3)
C7—C2—C3—C4	0.3 (3)	C15—O1—C1—C2	−179.96 (17)
C8—S1—C7—C2	177.93 (15)		

### Hydrogen-bond geometry (Å, º)

Cg is the centroid of the C2–C7 benzene ring.

**Table:**

*D*—H···*A*	*D*—H	H···*A*	*D*···*A*	*D*—H···*A*
C15—H15*C*···O2^i^	0.96	2.45	3.221 (3)	138
C12—H12···*Cg*^ii^	0.93	2.70	3.558 (2)	154

Symmetry codes: (i) −*x*+1, *y*−1/2, −*z*+3/2; (ii) −*x*+2, −*y*, −*z*+1.
